# Study on the relationship between soil aggregate size, electrical conductivity, organic carbon, and nutrient availability in highway slopes in alpine regions

**DOI:** 10.1371/journal.pone.0356363

**Published:** 2026-08-14

**Authors:** Mengke Zhu, Bocong Huang, Yingwei Ai, Zongyang Liu, Shenghao Ai, Meihua Sheng, Xiaoyan Ai

**Affiliations:** 1 Institute of Geographical Science, Henan Academy of Science, Zhengzhou, PR China; 2 Engineering Research of Eco-Mechanical Desertification Control and Ecosystem Carbon Sink, Ministry of Education, Chongqing Jiaotong University, Chongqing, PR China; 3 School of River and Ocean Engineering, Chongqing Jiaotong University, Chongqing, PR China; 4 Key Laboratory of Bio-Resource and Eco-Environment of Ministry of Education, College of Life Sciences, State Key Laboratory of Hydraulics and Mountain River Engineering, Sichuan University, Chengdu, PR China; 5 School of Civil Engineering and Geomatics, Southwest Petroleum University, Chengdu, China; 6 Chengdu Institute of Organic Chemistry, Chinese Academy of Sciences, Chengdu, PR China; 7 University of Chinese Academy of Sciences, Beijing, PR China; Harbin Normal University, CHINA

## Abstract

This study investigated the soil aggregate distribution on highway slopes in the alpine region of western Sichuan, China. The proportion of >5 mm aggregates was significantly higher than that of other size fractions (*P* < 0.05), whereas the content of 0.25–0.5 mm aggregates was the lowest. Across all aggregate size fractions, the contents of available nitrogen (AN), available phosphorus (AP), and available potassium (AK) were analyzed. Overall, the available nutrient contents in soil aggregates were relatively high at elevations of 3321 m and 3677 m, with AN and AP contents gradually decreasing as aggregate size increased. Further calculations revealed that the nutrient contribution rate of >5 mm aggregates was significantly higher than that of other size fractions (*P* < 0.05). This study also indicates that soil organic carbon (SOC) has a significant positive correlation with AP, and soil electrical conductivity (SEC) has a significant positive correlation with AN, AP, and AK. These findings provide new insights into the storage of available nutrients in highway slope soils in alpine regions and offer a theoretical basis for improving roadside ecosystems.

## 1. Introduction

As fundamental structural units of soil, aggregates play a pivotal role in maintaining soil fertility [1]. Soil aggregates physically protect and compartmentalize nutrients, simultaneously influencing their distribution patterns [2,3]. Studies show that nutrient distribution varies significantly across different aggregate size classes [4,5]. Sarker et al. (2018) highlighted the importance of finer aggregates in retaining nutrients under agricultural management [6]. Hu et al. (2020) experimentally confirmed that slope-scale erosion drives nutrient redistribution, likely leading to differences in nutrient availability among aggregate sizes [7]. Together, these findings reveal distinct nutrient distribution patterns across aggregate fractions and establish meaningful links between aggregate size and nutrient storage and bioavailability.

However, previous research has mainly concentrated on farmland and woodland soils, with limited attention given to nutrient distribution across aggregate fractions along altitudinal gradients in restored cut slopes of the southwestern subalpine mountains in China. In this region, winding road construction required extensive slope cutting along elevation gradients, significantly disrupting native ecosystems. Subsequent restoration efforts employed engineered soil mixtures to rehabilitate these slopes. In high-altitude mountainous regions, the long-term stability of cut slopes is governed not only by ecological restoration but also by underlying geotechnical and geomorphological conditions [8,9]. Moreover, the engineering characteristics of solidified soils are highly relevant to the design and implementation of composite soil mixtures used in slope stabilization [10]. Soil structure and nutrient availability govern the long-term stability of slope restoration. These geotechnical considerations provide an essential foundation for understanding the soil structural and nutrient dynamics examined in this study. The subalpine environment is a fragile ecosystem marked by persistent low temperatures, high UV radiation, and reduced oxygen levels [11]. Existing studies indicate that freeze–thaw cycles and human disturbances affect soil aggregate formation and nutrient cycling [12,13]. Under varying environmental conditions-such as temperature and precipitation-and ongoing soil development processes, aggregate size distributions gradually change. Although our earlier research on these restored slopes examined nutrient content, aggregate stability, and soil quality [14], a systematic evaluation of nutrient distribution across different aggregate size fractions is still needed.

SOC and soluble salts are key factors regulating soil fertility and nutrient availability. As a direct source of nutrients, SOC also indirectly promotes nutrient release and retention by improving soil structure and stimulating microbial activity [15]. SEC, which reflects the concentration of soluble salts in soil, directly affects ionic balance, osmotic pressure, and nutrient transport in the soil solution, thereby significantly influencing the bioavailability of available nutrients such as nitrogen, phosphorus, and potassium [16]. This relationship is especially critical in the ecologically fragile high-altitude cold regions along highway slopes, where soil nutrient cycling is more sensitive and complex due to engineering disturbances and extreme climatic conditions. However, current research on the relationships between SOC and SEC and soil available nutrients remains insufficient.

**Fig 1:**
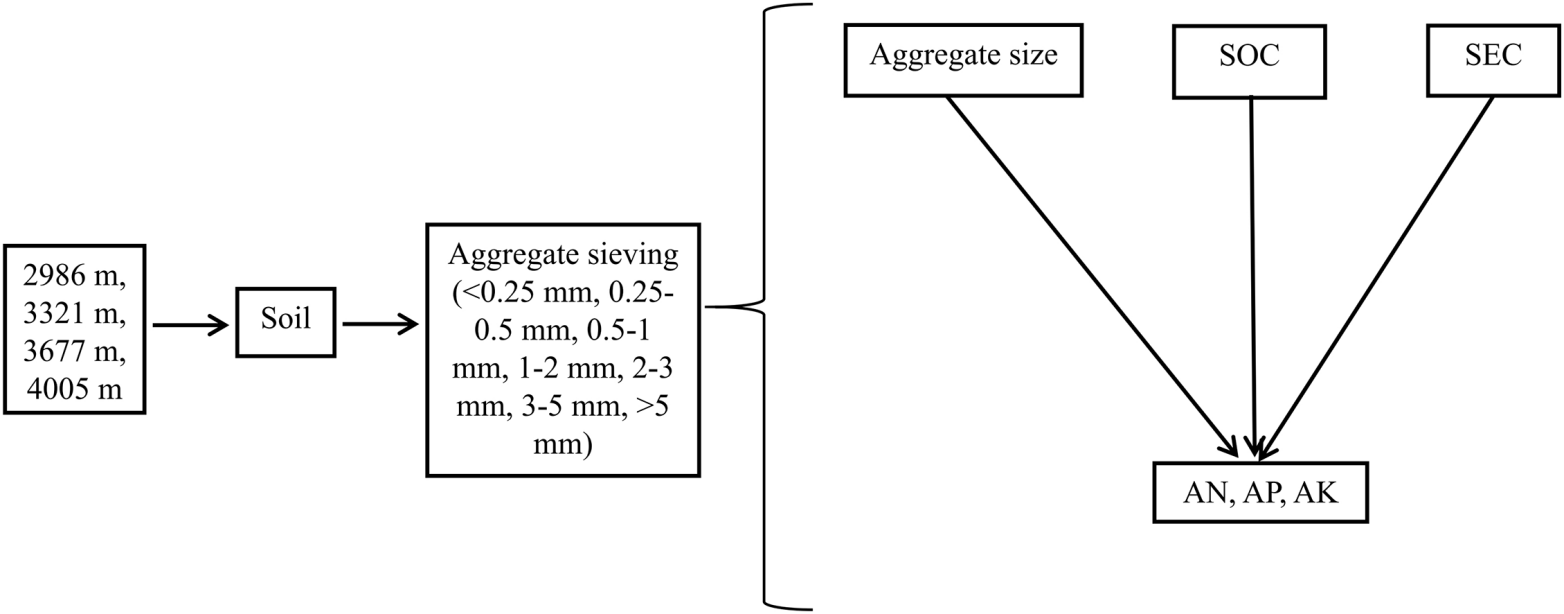
The conceptual figure of this article.

Successful ecological reconstruction of cut slopes necessitates careful consideration of both soil structural integrity and nutrient retention capacity, which are critical determinants of restoration outcomes. This paper raises the following question: (1) Given the differences in the distribution and nutrient content of aggregates of different sizes, do their contributions to soil nutrients differ significantly? (2) How do SOC and SEC influence the content of available nutrients? (Fig 1). A comprehensive study on the nutrient storage dynamics across aggregate size classes, their contribution patterns, and the effects of SOC and SEC on available nutrients will provide valuable insights for soil nutrient retention and supply in highway slope soils of high-altitude cold regions, integrating both soil physical and chemical perspectives.

## 2. Materials and methods

### 2.1. Study site characterization

**Fig 2:**
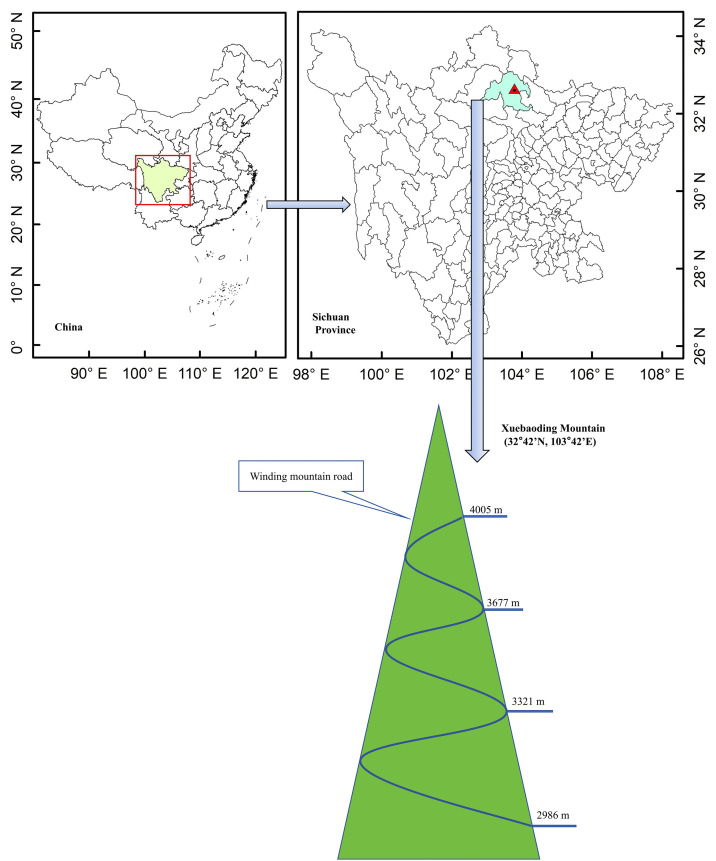
Schematic diagram of sampling sites along the altitude.

Xuebaoding Mountain, the highest peak in the region, reaches an elevation of 5588 m (32°42′N, 103°42′E). As shown in Fig 2, the study area is located in Songpan County, Aba Tibetan and Qiang Autonomous Prefecture, Sichuan Province, China. This region experiences a humid subtropical monsoon climate. After highway construction, the cut slopes were ecologically restored using a composite soil mixture-composed of native soil, water-retaining agents, organic fibers, binding materials, and fertilizers-which was applied to the slope surfaces via hydraulic spraying. The specific composition of the artificial soil mixture used for the highway slopes is shown in Table 1 [17]. This slope stabilization method improves geotechnical stability while creating favorable conditions for ecological recovery. The soil texture is Loamy Sand at 2986 m; The soil texture is Sandy Loam at 3321 m, 3677 m and 4005 m.

**Table 1:** Composition of artificial soil matrix with outsoil spraying technology.

Mixed materials	Proportions
Humus	6.25 kg/m^2^
Straw	0.1 kg/m^2^
Soluble chemical fertilizer	(N: P2O5: K2O = 16: 6: 8) 0.3 kg/ m^2^
Composite materials (binders and water-retaining agents)	0.225 kg/m^2^
Seeds (perennial ryegrass, alfalfa, etc.)	0.03 kg/m^2^
Backfill soil (rock fragments and agricultural soil)	Local native soil

### 2.2. Sampling design and soil collection

Prior to sampling, permission for field access was obtained from the Aba Tibetan and Qiang Autonomous Prefecture Transportation Bureau. No additional permits were required as the sampling sites were located within public highway rights-of-way and did not involve protected areas or endangered species. All soil samples were collected from the 0–10 cm surface layer in October 2017. Sampling covered four elevation treatments (2986 m, 3321 m, 3677 m, and 4005 m; Fig 2), with three replicates per elevation. A total of 12 soil samples were collected. Vegetation types varied with elevation: deciduous broadleaf forest dominated at 2986 m, coniferous forest prevailed at 3321 m, shrubland was predominant at 3677 m, and herbaceous vegetation characterized the 4005 m zone. Each composite sample was collected following an “S”-shaped sampling pattern, consisting of 20–25 systematically taken subsamples that were thoroughly mixed. After removing plant residues and rock fragments, the samples were bagged, labeled, and transported to the laboratory for analysis.

### 2.3. Soil analysis

Soil aggregates were separated by dry sieving and categorized into seven size classes: < 0.25 mm, 0.25–0.5 mm, 0.5–1 mm, 1–2 mm, 2–3 mm, 3–5 mm, and >5 mm [18]. The mass percentage of each aggregate fraction was calculated using the following equation:


$$\begin{array}{c} P(\%)=\frac{m_{i}}{\sum_{i=1}^{n}m_{i}} \end{array}$$ (1)

where ***P*** is the mass percentage of each aggregate size class, ***mᵢ*** denotes the mass of a specific size class, and **n** is the total number of size classes (here, n = 7).

AN was determined by the alkaline hydrolysis diffusion method, AP by the Olsen extraction method, AK by flame emission spectrophotometry, SOC by potassium dichromate oxidation with external heating, and SEC with a DDS-307 conductivity meter (Shanghai Yueping Scientific Instrument Company, China).

The relative nutrient contribution (C, %) for each aggregate size fraction was determined by the following equation:


$$\begin{array}{c} C(\%)=\frac{m_{i}a_{i}}{\sum_{i=1}^{n}m_{i}a_{i}} \end{array}$$ (2)

where ***C*** is the nutrient contribution rate of an aggregate size class, ***mᵢ*** and ***aᵢ*** denote its mass and available nutrient content, respectively, and **n** is the total number of size classes (here, n = 7).

### 2.4. Statistical analysis

A completely randomized design was adopted with four elevation treatments (2986 m, 3321 m, 3677 m, and 4005 m) and three replicates per treatment (n = 3). One-way analysis of variance (ANOVA) was performed using OriginPro 8.0 to compare the means of soil properties among different aggregate size fractions or across elevations. Least significant difference (LSD) tests were used for multiple comparisons, and statistical significance was set at *P* < 0.05. OriginPro 8.0 was employed to generate scatter plots illustrating the relationships between soil available nutrient contents and aggregate size fractions, and to carry out Pearson correlation and regression analyses.

## 3. Results

### 3.1. Variation in available nutrient content across aggregate-size fractions

**Fig 3:**
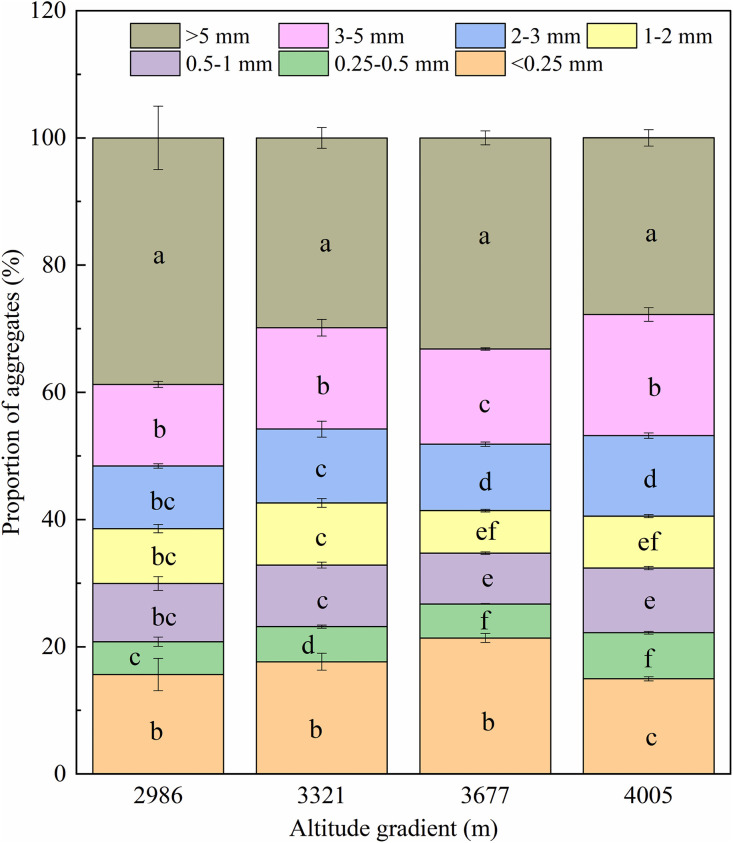
Aggregate size distribution across altitudes. The chart shows variations in the percentage of soil aggregate size fractions. Lowercase letters are used to indicate statistically significant differences (LSD test, *P* < 0.05) among the cut slopes at each altitude level.

As shown in Fig 3, the 0.25–0.5 mm aggregate fraction was consistently the least abundant, whereas the > 5 mm fraction was significantly more abundant than all other size classes (*P* < 0.05) across the four elevation gradients. At 2986 m, 3321 m, and 3677 m, abundance followed the decreasing order: > 5 mm> <0.25 mm > 3−5 mm > 2−3 mm. At 4005 m, the sequence was: > 5 mm > 3−5 mm> <0.25 mm > 2−3 mm > 0.5−1 mm > 1−2 mm > 0.25–0.5 mm.

**Fig 4:**
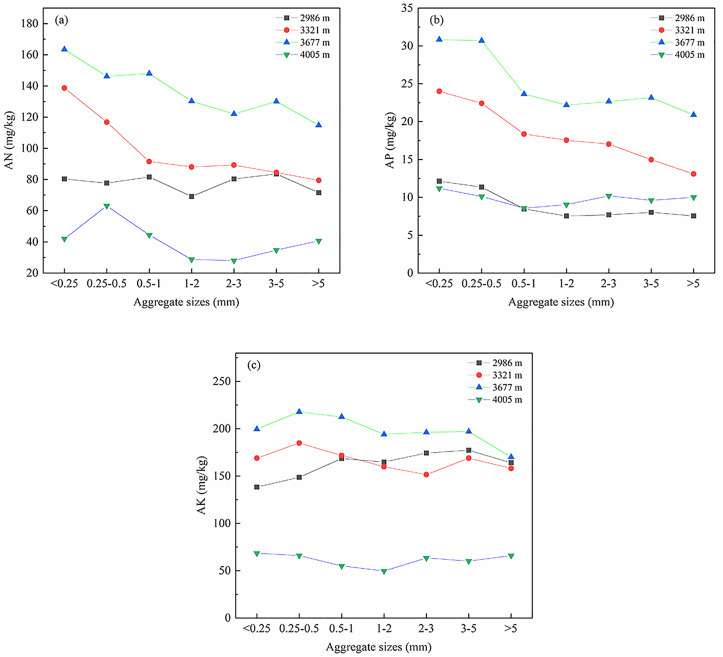
Variation of available nutrients in soil aggregates at different altitudes. AN: available nitrogen; AP: available phosphorus; AK: available potassium.

As illustrated in Fig 4, the distribution of available nutrients varied markedly with elevation. At 2986 m, AN peaked in 3−5 mm aggregates (Fig 4a), AP in <0.25 mm aggregates (Fig 4b), and AK also in 3−5 mm aggregates (Fig 4c). At 3321 m, both AN and AP reached their maxima in <0.25 mm aggregates (Fig 4a, 4b), while AK was highest in the 0.25–0.5 mm fraction (Fig 4c). At 3677 m, AN and AP concentrations were again highest in <0.25 mm aggregates (Fig 4a, 4b), with AK peaking in 0.25–0.5 mm aggregates (Fig 4c). At 4005 m, the 0.25–0.5 mm fraction exhibited the highest AN content (Fig 4a). Cross-elevation comparison revealed that concentrations of AN, AP, and AK were consistently highest at 3677 m (Fig 4). AP levels at 3321 m were notably higher than those at 2986 m and 4005 m (Fig 4b). Conversely, both AN and AK at 4005 m were consistently lower than at all other elevations (Fig 4a, 4c). For AN, AP and AK, both altitude and aggregate size had significant main effects (*P* < 0.05), and their interaction was also significant (*P* < 0.05) (Table 3).

**Table 3:** The results of the two-factor analysis of variance.

	AN		AP		AK		SOC		SEC	
	F value	P value	F value	P value	F value	P value	F value	P value	F value	P value
Altitude	205.156	0.000	247.494	0.000	519.159	0.000	429.801	0.000	33.057	0.000
Aggregate sizes	9.750	0.000	16.338	0.000	2.572	0.029	8.710	0.000	3.368	0.007
AltitudeⓍAggregate sizes	2.296	0.009	2.128	0.016	3.312	0.000	5.040	0.000	0.607	0.879

Notes: AN: available nitrogen; AP: available phosphorus; AK: available potassium; SOC: soil organic carbon; SEC: soil electrical conductivity.

### 3.2. Contribution rates of available nutrients across aggregate-size fractions

**Fig 5:**
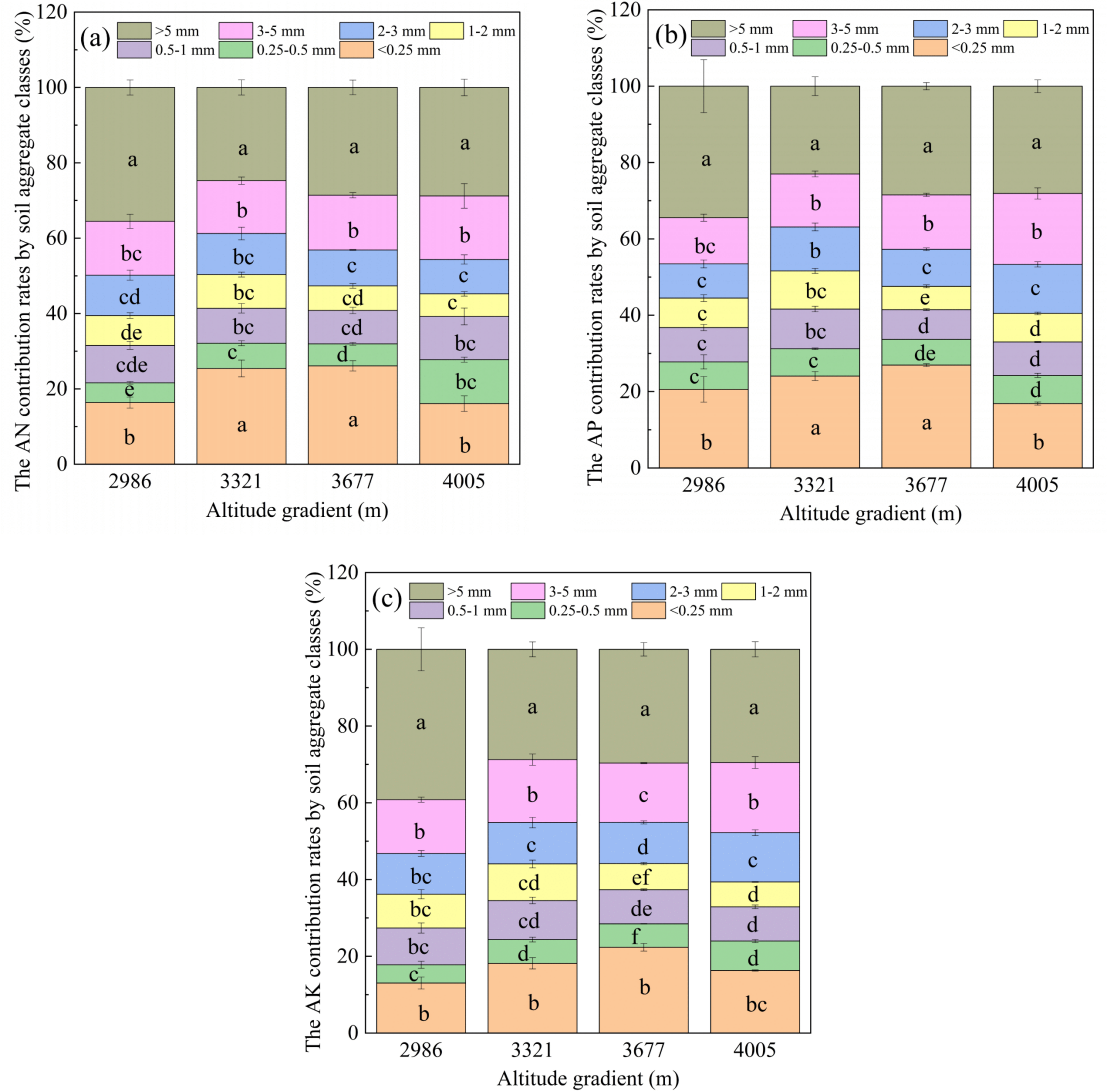
Contribution rates of AN, AP, and AK across soil aggregate classes at different altitudes. Lowercase letters show significant differences (LSD test, *P* < 0.05) in aggregate size distribution among classes within each altitude.

Fig 5 presents the contribution rates of available nutrients across soil aggregate size fractions. Three aggregate fractions (>5 mm, < 0.25 mm, and 3–5 mm) consistently exhibited the highest contribution to AN (Fig 5a), AP (Fig 5b), and AK (Fig 5c), irrespective of elevation. At 2986 m, 3321 m, and 3677 m, the 0.25–0.5 mm and 1–2 mm fractions showed relatively low AN contributions; at 4005 m, the 1–2 mm and 2–3 mm fractions exhibited the lowest AN contribution (Fig 5a). Across all elevation gradients, the 0.25–0.5 mm and 1–2 mm fractions displayed the lowest contribution rates to both AP (Fig 5b) and AK (Fig 5c).

### 3.3. The relationship between SOC and SEC with available nutrients

**Fig 6:**
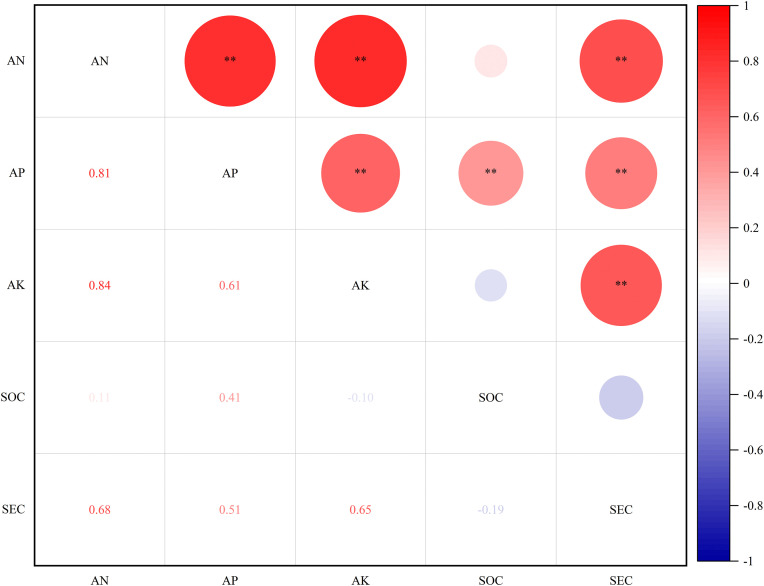
Results of the Pearson correlation analysis for soil aggregate indicators. A double asterisk (**) indicates a significant correlation at the *P* < 0.01 level.

**Fig 7:**
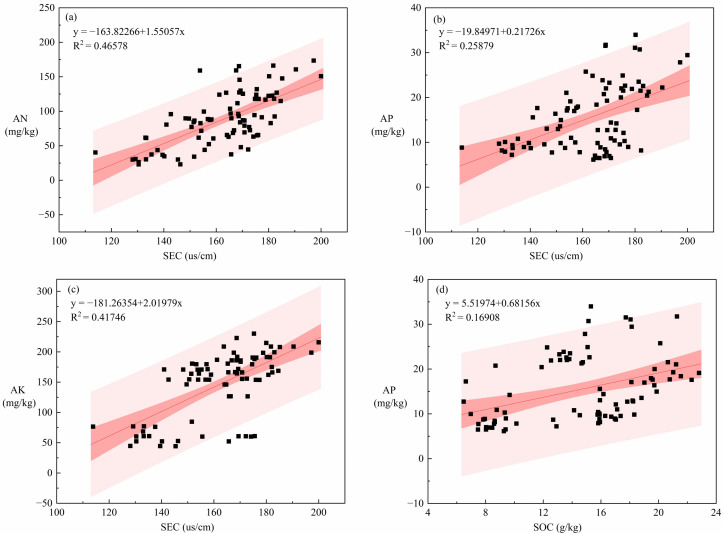
Regression analysis of SEC, SOC, and available nutrient indicators. AN: available nitrogen; AP: available phosphorus; AK: available potassium.

Table 2 presents the SOC and SEC measurements across different aggregate size fractions. The highest SOC content was found in the 0.25–0.5 mm aggregates at 2986 m, in the 0.5−1 mm aggregates at 3321 m, and again in the 0.25–0.5 mm aggregates at 3677 m-where it was significantly greater than in all other size classes (*P* < 0.05). At 4005 m, the < 0.25 mm aggregates showed the highest SOC content. In terms of SEC, no significant differences were observed among the seven aggregate fractions at 2986 m, 3677 m, and 4005 m (*P* > 0.05). At 3321 m, however, the 0.25–0.5 mm aggregates recorded the highest SEC. For SOC, both factors demonstrated significant main effects, and there was a significant interaction (*P* < 0.05). For SEC, both factors also showed significant main effects (*P* < 0.05), while the interaction between altitude and aggregate size was not significant (Table 3). Correlation analysis revealed that SOC was significantly and positively correlated with AP (*P* < 0.01), whereas SEC showed significant positive correlations with AN, AP, and AK (*P* < 0.01) (Fig 6). Regression analysis further indicated that increasing SEC was associated with rising trends in AN, AP, and AK (Fig 7a-7c), while increasing SOC corresponded to an upward trend in AP (Fig 7d). Taken together, these results suggest that SEC serves as an important regulator of soil available nutrients, while SOC is a key factor specifically influencing AP.

**Table 2:** SOC and SEC contents within soil aggregates of different sizes.

	altitude gradient (m)	Aggregate sizes (mm)						
		<0.25	0.25-0.5	0.5-1	1-2	2-3	3-5	>5
SOC (g/kg)	2986 3321 3677 4005	6.86 ± 0.31Db 21.17 ± 0.08Aab 15.12 ± 0.12Cb 17.76 ± 0.35Ba	9.28 ± 0.27Da 20.17 ± 0.27Abc 17.98 ± 0.13Ba 17.15 ± 0.11Cab	8.76 ± 0.30Ca 22.22 ± 0.37Aa 14.10 ± 0.50Bbc 13.84 ± 1.04Bd	8.92 ± 0.63Ca 19.76 ± 0.53Abc 13.81 ± 0.45Bbc 15.55 ± 0.53Bbc	7.83 ± 0.16Dab 19.14 ± 0.51Acd 13.34 ± 0.40Cbc 16.20 ± 0.26Babc	8.31 ± 0.24Ca 17.33 ± 1.27Ad 13.55 ± 0.87Bbc 16.21 ± 0.38Aabc	8.54 ± 0.79Ba 18.42 ± 0.20Acd 11.78 ± 1.75Bc 15.28 ± 0.57 cd
SEC (us/cm)	2986 3321 3677 4005	171.00 ± 4.90ABa 163.97 ± 5.09Bab 186.40 ± 5.42Aa 158.87 ± 5.39Ba	173.70 ± 1.95Aa 170.43 ± 4.62Aa 182.90 ± 9.15Aa 161.03 ± 13.87Aa	176.17 ± 3.49Aa 158.30 ± 3.51Bab 177.77 ± 6.70Aa 137.57 ± 2.29Ca	164.23 ± 2.96Ba 157.53 ± 0.40Bab 176.73 ± 4.44Aa 134.63 ± 5.43Ca	161.97 ± 6.93Ba 148.77 ± 3.29BCb 181.10 ± 1.25Aa 136.23 ± 5.16Ca	163.43 ± 5.07Aa 154.50 ± 8.72Aab 169.63 ± 3.60Aa 144.57 ± 10.77Aa	162.27 ± 2.56ABa 158.20 ± 7.21ABab 178.27 ± 5.13Aa 140.47 ± 17.03Ba

Notes: SOC: soil organic carbon; SEC: soil electrical conductivity.

## 4. Discussion

### 4.1. Soil aggregate distribution across different particle sizes

Previous studies have reported similar patterns in aggregate size distribution and its influence on soil processes. Wang et al. (2019) observed that in tea plantations of varying ages, the > 5 mm fraction predominated, followed by the < 0.25 mm fraction [19]. Likewise, Lu et al. (2019) noted a higher proportion of macroaggregates (>0.25 mm) compared to microaggregates (<0.25 mm) in a temperate fen ecosystem [20]. These findings are consistent with the results of the present study, in which the > 5 mm fraction consistently exhibited significantly higher proportions than other size classes across all elevations. Such differences may stem from variations in soil types, including the presence of binding agents that enhance macroaggregate formation and physical processes that favor larger structural units.

The distribution ratio and developmental degree of different soil aggregate size fractions are considered indicators of climate change, while also being affected by climatic variations [21]. This study was conducted in the subalpine mountains of southwest China, where perennial low temperatures and frequent freeze-thaw cycles likely favor the formation and preservation of large aggregates (>5 mm). In addition, human activities are known to significantly alter aggregate stability and size distribution [22,23]. Previous research has shown that fertilizer application-including chemically balanced formulations-can substantially modify the proportions of aggregate size fractions [24]. Given that the soils studied contained engineered mixtures with fertilizers, the observed aggregate distribution may partly reflect such anthropogenic inputs. Soil aggregate formation is closely linked to soil chemical properties [25]. Multiple internal mechanisms regulate aggregate size distribution, with studies reporting positive correlations between aggregate size and cementing agents such as organic matter, clay minerals, and their complexes [26]. In slope restoration projects using engineered soil mixtures, the inclusion of binding agents for soil stabilization and erosion control likely further promotes the formation of macroaggregates (>5 mm).

Artificial additive-driven aggregation (short-term, rapid). The engineered soil mixture applied during restoration contains polymeric binding agents, organic fibers, and cementitious materials specifically designed for rapid slope stabilization. These additives likely promote the formation of macroaggregates (>5 mm) through direct physicochemical mechanisms, including polymer bridging between soil particles, electrostatic interactions, and mechanical entanglement of fibers. This pathway is expected to dominate aggregate size distribution during the initial years post-restoration. Natural soil-forming aggregation (long-term, gradual). Concurrent with the rapid effects of artificial additives, natural processes begin to contribute to aggregate formation and stabilization over longer timescales. These include: (i) physical processes such as freeze–thaw cycling, which can both disrupt and re-form aggregates; (ii) biochemical processes including the accumulation of soil organic matter from vegetation litter and root exudates, which serve as natural binding agents; (iii) biological processes involving fungal hyphae, bacterial extracellular polymeric substances, and root networks that physically entangle and stabilize aggregates [27]. While our data cannot fully separate these contributions, the proposed model provides a conceptual framework for future investigations aimed at understanding aggregate dynamics in restored ecosystems.

### 4.2. Concentrations of available nutrients across soil aggregate fraction

Aggregates exhibit size-dependent differences in their capacity to adsorb and release trace elements [28]. In agricultural systems, reduced soil disturbance and increased crop residue inputs are critical for retaining organic carbon and nutrients within finer aggregate fractions [6]. Our results show higher concentrations of AN and AP in smaller aggregates (<0.5 mm) at 3321 m and 3677 m, aligning with the reported tendency for finer fractions to store more nutrients. Regarding phosphorus dynamics, findings vary: some studies report greater P adsorption in larger aggregates of highly weathered soils, while others show preferential P retention in smaller fractions (<0.5 mm) [29]. For potassium, Liu et al. (2019) found that K⁺ (the dominant form in soil) receives limited protection within aggregates [30]; however, Yu et al. (2017) demonstrated that aggregates with a higher specific surface area-a feature typical of smaller fractions—retain mineral nutrients more effectively [31]. Our data support the view that smaller aggregates (<0.5 mm) promote higher nutrient bioavailability, likely because their greater specific surface area enhances their ability to adsorb and retain available nutrients.

The observed variation patterns of AN, AP and AK within the altitude range may reflect the changes in soil C:N:P chemical stoichiometry driven by vegetation types and freeze-thaw dynamics. These stoichiometric changes regulate the retention or loss of nutrients on the recovery slopes [32]. Previous studies have shown that freeze–thaw cycles can significantly affect soil nutrient dynamics [33]. In high-altitude cold regions such as our study area, frequent freeze-thaw cycles likely play a critical role in redistributing nutrients among aggregate size fractions. We propose that the observed enrichment of available nutrients in smaller aggregates (<0.5 mm) at mid-elevations (3321 m and 3677 m) can be attributed to the following interrelated physicochemical mechanisms.

First, physical expulsion during aggregate disruption. When soil freezes, the volumetric expansion of water generates mechanical stresses within soil pores. Repeated freeze-thaw cycles cause the fragmentation of macroaggregates, particularly through the collapse of macropores and the weakening of interparticle bonds. This disruption expels pore water containing dissolved nutrients into the surrounding soil matrix. Smaller aggregates, with their higher capillary forces and surface-area-to-volume ratio, are more effective at retaining these expelled solutes, leading to nutrient enrichment in finer fractions. Second, chemisorption on newly exposed mineral surfaces. Aggregate breakdown during freezing and thawing exposes fresh mineral surfaces-including clay minerals, iron and aluminum oxides, and organic matter-that were previously encapsulated within macroaggregate interiors [34]. These newly exposed surfaces possess abundant reactive sites that can strongly adsorb nutrient ions such as NH_4_ ^+^ , K^+^ , and PO_4_^3^⁻ via chemisorption or electrostatic interactions. This mechanism is particularly relevant for phosphorus, which tends to form inner-sphere complexes with metal oxides. Third, concentration by ice lens formation and solute migration. During freezing, ice lens formation creates thermal and hydraulic gradients that drive the migration of soil water toward the freezing front. As water freezes, solutes are excluded from the ice crystal structure and become concentrated in the unfrozen water films surrounding ice lenses. Finer aggregate fractions, due to their higher water retention capacity and stronger capillary forces, act as preferential sinks for these concentrated solutes. Upon thawing, the accumulated nutrients remain retained in these finer fractions.

These three mechanisms likely operate synergistically in our study system, contributing to the nutrient enrichment observed in smaller aggregates. Notably, this enrichment was most pronounced at mid-elevations (3321 m and 3677 m), where moderate freeze–thaw intensity may optimize aggregate disruption and solute redistribution without causing excessive leaching. At lower elevation (2986 m), coarser soil texture and warmer conditions may promote nutrient leaching rather than retention [14]. At the highest elevation (4005 m), persistent snow cover may insulate soils from frequent freeze-thaw cycles, or extreme freeze–thaw intensity may accelerate aggregate turnover and nutrient loss. Together, these mechanisms account for the higher available nutrient concentrations in smaller aggregates at mid-elevations and the lower nutrient availability observed at the extremes of the elevation gradient.

### 4.3. Contribution rates of nutrients among soil aggregate fractions

Our study revealed that different aggregate size fractions contribute unevenly to soil nutrient pools. The > 5 mm fraction showed the highest contribution rates, consistent with findings reported in grassland and orchard ecosystems [35]. This result aligns with established knowledge that nitrogen accumulation primarily occurs in macroaggregates, which constituted the dominant fraction in our study [36]. Wang et al. (2019) also observed elevated nutrient concentrations in both >5 mm and <0.25 mm fractions in tea plantation soils [19]. Although the > 5 mm aggregates had lower available nutrient concentrations, their large mass proportion across the elevation gradient translated into a significant nutrient contribution. Conversely, the < 0.25 mm fraction, despite its lower abundance, contributed substantially due to its higher nutrient content. These results indicate that contributions to nutrient pools depend on both the abundance of aggregates and their inherent nutrient concentrations, implying that restoration strategies could enhance nutrient retention by intentionally improving soil structure. While this study offers valuable insights, certain limitations should be noted, such as single-site sampling and a limited time frame. It is important to note, however, that in erosion-prone highway cut slopes, the physical stability of aggregates is a critical factor determining whether the nutrients they contain are retained in situ or lost via surface runoff and sediment transport. While the > 5 mm fraction contributed the most to the nutrient pool due to its large mass proportion, its relatively low nutrient concentration and potentially higher susceptibility to physical displacement under rainfall or freeze–thaw conditions may limit its role as a stable long-term nutrient reservoir. Previous studies have shown that macroaggregates can be preferentially removed by overland flow on steep slopes, particularly when soil structure is disturbed. Therefore, the contribution rates reported here reflect the static nutrient distribution at the time of sampling, but should be interpreted with caution when inferring nutrient retention capacity. Future investigations integrating aggregate stability assessments with erosion monitoring are needed to clarify the dynamic balance between nutrient contribution and physical stability in such vulnerable slope environments. Future research should adopt longitudinal, multi-scale monitoring to better understand the spatiotemporal patterns of aggregate-associated nutrient dynamics in restored cut slopes.

### 4.4. The relationship between SEC, SOC and available nutrients

Correlation and regression analyses underscore the distinct regulatory roles of SEC and SOC in soil fertility. Strong positive correlations and corresponding regression trends indicate that SEC is a key factor enhancing the bioavailability of multiple essential nutrients-AN, AP, and AK. However, this finding warrants careful interpretation given that the study sites were restored using engineered soil mixtures containing fertilizers, organic fibers, and binding materials. These amendments directly introduce soluble salts (contributing to SEC) and available nutrients simultaneously, which may partially account for the observed positive correlations. Therefore, the SEC–nutrient relationships reported here should be understood as the net outcome of combined factors, including restoration inputs, natural soil development, and site-specific environmental processes, rather than an inherent soil property independent of management history. This may be explained by the role of soluble salts in moderating soil acidity, reducing phosphorus and potassium fixation, and consequently improving their solubility and availability in the soil solution. The soluble salts in our study sites are likely dominated by nutrient cations (e.g., K ⁺ , NH_4_ ⁺ , Ca^2+^) rather than harmful Na ⁺ . Although soluble cations typically bind readily with phosphate to form precipitates that limit phosphorus availability [37], our study reached a contrary conclusion. One possible explanation is that freeze–thaw cycles in high-altitude roadside slopes disrupt soil aggregates, releasing occluded cations (e.g., Ca^2+^ , Mg^2+^ , K ⁺ , NH_4_⁺) and organic or inorganic phosphorus. Alternatively, intense soil mineralization in this environment may simultaneously elevate soluble salts and available nutrients. In contrast, SOC shows a specific and strong positive association only with AP. This suggests that SOC may influence phosphorus availability directly-through the release of organic anions that compete with phosphate for adsorption sites-or indirectly, by stimulating microbial activity that promotes phosphorus mineralization [38].

It is important to note that the correlations reported here do not imply direct causal relationships between SEC, SOC, and available nutrients. Several unmeasured or partially characterized factors may contribute to the observed associations. For instance, the intensity of soil weathering and organic matter mineralization can simultaneously release soluble salts and available nutrients, particularly in high-altitude environments where freeze–thaw cycles promote aggregate disruption and nutrient mobilization. Soil moisture regime, which varies with elevation and slope position, may also influence both SEC (via concentration or dilution of soluble salts) and nutrient availability. Soil pH, which was not measured in this study, can play an important role in regulating phosphorus availability. Therefore, the possibility that the observed positive SOC-AP relationship may be partially mediated by pH-related effects cannot be ruled out. Future studies should incorporate pH measurements to better disentangle the direct and indirect mechanisms underlying SOC-mediated phosphorus availability. We have also slightly moderated the language regarding the mechanistic interpretation of the SOC-AP link, emphasizing that the proposed mechanisms (anion competition and microbial mineralization) are plausible based on existing literature, but should be considered with the caveat that pH-related effects were not examined in this study. Furthermore, the use of engineered soil mixtures containing fertilizers and binding agents introduces inherent spatial heterogeneity in both salt content and nutrient pools, potentially generating correlations that do not reflect mechanistic linkages. Future studies incorporating direct measurements of mineralization rates, moisture dynamics, and parent material composition are needed to disentangle these interacting factors and establish causal pathways.

## 5. Conclusions

This study quantified the contributions of different soil aggregate size fractions to available nutrients on highway cut slopes in the high-altitude region of western Sichuan, and further analyzed the relationships of SEC and SOC with nutrient availability. In mid-elevation zones (3321 m and 3677 m), smaller aggregate fractions (<0.5 mm) exhibited a greater capacity for nutrient retention. The > 5 mm fraction dominated the aggregate distribution and therefore contributed the most to the overall nutrient pool among all size fractions. Soil electrical conductivity showed significant positive correlations with multiple available nutrients, whereas soil organic carbon exhibited a strong positive correlation only with available phosphorus. These findings provide a valuable reference for assessing the soil nutrient storage potential of highway slopes in high-altitude regions.

## Supporting information

S1 Data Minimal dataset. (XLSX)
